# Systematic Review of Somatic Mutations in Splenic Marginal Zone Lymphoma

**DOI:** 10.1038/s41598-019-46906-1

**Published:** 2019-07-18

**Authors:** Carolina Jaramillo Oquendo, Helen Parker, David Oscier, Sarah Ennis, Jane Gibson, Jonathan C. Strefford

**Affiliations:** 1 Genomic Informatics, Human Genetics and Genomic Medicine, Faculty of Medicine, University of Southampton, Southampton, United Kingdom; 2 Cancer Sciences, Faculty of Medicine, University of Southampton, Southampton, UK; 3 Department of Haematology, Royal Bournemouth Hospital, Bournemouth, UK

**Keywords:** B-cell lymphoma, Cancer genomics, Molecular medicine, Next-generation sequencing

## Abstract

The aims of this systematic review are to refine the catalogue of somatic variants in splenic marginal zone lymphoma (SMZL) and to provide a well-annotated, manually curated database of high-confidence somatic mutations to facilitate variant interpretation for further biological studies and future clinical implementation. Two independent reviewers systematically searched PubMed and Ovid in January 2019 and included studies that sequenced SMZL cases with confirmed diagnosis. The database included fourteen studies, comprising 2817 variants in over 1000 genes from 475 cases. We confirmed the high prevalence of *NOTCH2, KLF2* and *TP53* mutations and analysis of targeted genes further implicated *TNFAIP3*, *KMT2D*, and *TRAF3* as recurrent targets of somatic mutation based on their high incidence across studies. The major limitations we encountered were the low number of patients with whole-genome, unbiased analysis and the relative sensitivities of differing sequencing approaches. Overall, we showed that there is little concordance between whole exome sequencing studies of SMZL. We strongly support the continuing unbiased analysis of the SMZL genome for mutations in all protein-coding genes and provide a valuable database resource to facilitate this endeavour that will ultimately improve our understanding of SMZL pathobiology.

## Introduction

The ability to perform unbiased massively-parallel sequencing (MPS) of tumour and germ-line DNA pairs, has allowed cancer researchers to catalogue the mutational landscape of human tumours. This has offered important mechanistic insights into the biology of cancer initiation, progression and drug resistance that promises to transform the diagnosis, management and prevention of cancer. However, these important insights have not been made for rare tumour types, excluded from publicly available initiatives such as the International Cancer Genome Consortium (ICGC) and The Cancer Genome Atlas (TGCA). Mature B-cell malignancies are a heterogeneous group of diseases that arise during different stages of B-cell differentiation. Follicular lymphoma (FL), chronic lymphocytic leukaemia (CLL) and diffuse large B-cell lymphoma (DLBCL) have been at the heart of the ICGC project. Other less prevalent lymphomas such as those of marginal zone origin, namely nodal marginal zone lymphoma (NMZL), extranodal marginal zone lymphoma (MALT) and splenic marginal zone lymphoma (SMZL) are currently excluded from the ICGC and other international consortia, resulting in limited genomic data for these rarer cancers.

SMZL comprises approximately 2% of all lymphoid tumours and is classified by the World Health Organization (WHO) as a low grade, indolent B-cell neoplasm involving the spleen, bone marrow and frequently peripheral blood. The median age at diagnosis is 65 years. Patients usually present either with abdominal discomfort secondary to splenomegaly, symptoms of anaemia or incidentally due to asymptomatic splenomegaly or an abnormal blood count. Median survival is approximately 10 years; 70% of patients present with or develop progressive disease with a requirement for therapy, and 5–10% will undergo transformation to a large B-cell lymphoma. Patients often respond well to first-line treatment with splenectomy or rituximab-based regimes, but approximately 30% of patients have a poor outcome. Whilst, definitive diagnosis still requires splenic histology, in practice, the indications for splenectomy are diminishing and diagnosis is frequently based on a combination of clinical features, lymphocyte morphology, bone marrow histology, immunophenotype and the absence of features that define related disorders. An example is the provisional WHO entity, splenic diffuse red pulp lymphoma (SDRPL) which is often difficult to distinguish from SMZL in the absence of splenic histology. Further diagnostic support may come from both immunoglobulin variable chain gene (*IGHV*) sequencing and cytogenetic analysis. The IGHV1–2*04 gene is used in approximately 30% of SMZL cases but not in related disorders, while deletions of the long arm of chromosome 7 (del7q) also occur in 30–40% of SMZL but rarely in other B cell lymphoproliferative disorders associated with splenomegaly.

Whole genome (WGS)^1^, exome (WES)^2–6^ and targeted sequencing analysis^7–14^, have discovered a number of recurrently mutated genes in SMZL. Whilst the number of cases analysed with these approaches remains relatively low, the reported somatic mutations preferentially target physiologically important processes such as cell cycle control and the differentiation or trafficking of marginal zone B-cells. The most significant mutations in SMZL target *KLF2* (20–40% of cases)^6,9,10^, *NOTCH2* (10–20%)^1–3,9^ and *TP53* (10–15%) and are supported by extensive genomic analysis, and preliminary functional work^1–3,6,9,10^. A panel of less prevalent mutations have also been reported targeting key biological pathways, though their prevalence is uncertain, and their importance remains opaque.

Only six genome-wide studies of somatic mutations in SMZL have been published to date. The only WGS study, published by Kiel and colleagues was limited to six cases, without matched germ-line DNA^1^. Rossi *et al*.^2^ employed WES to profile tumour and germ-line DNA from eight discovery cases with subsequently targeted relevant genes in additional samples. Four other WES studies have also been reported, analysing tumour and germ-line DNA from two, three, seven and fifteen cases^2–6^. Targeted re-sequencing approaches have also been applied, often with limited analysis of germ-line material, to identify mutations in genes that have a known or postulated role in SMZL or more generally B-cell tumour pathogenesis. Taken together, these studies provide a partial overview of the biological pathways targeted by somatic mutations in SMZL. However, several factors limit these studies; (1) the limited number of patients; (2) the clinical and biological heterogeneity of published cohorts; (3) the lack of available germ-line DNA from historical series; (4) the biased nature of targeted re-sequencing approaches, and (5) the variable experimental and bioinformatics methods. Furthermore, not only is there a limited number of patients analysed with unbiased WGS/WES, there is a striking lack of concordance between these studies, suggesting that significantly more cases need to be profiled^6^. This paucity of data has resulted in a lack of annotated information on somatic variants in the public domain.

This paper reports a systematic review of the previously identified somatic variants in SMZL, producing a single database of variants with consistent and detailed annotation. Given the preclusion of SMZL from international sequencing consortia, and the consequent lack of annotated information on public databases, our work represents an important, initial step in further facilitating the interpretation of somatic gene mutation data from SMZL. The final database is comprised of over 1000 genes containing 2817 variants in 475 samples. We summarise the data to highlight the recurrent genes and variants in SMZL creating a representative overview of the published SMZL data to date. While the analysis did emphasise the importance of well-established genes in SMZL (*KLF2* and *NOTCH2*) the pooled data allowed the identification of less studied genes (*TNFAIP3, KMT2D, IGLL5 and MYD88*), hotspots and recurrent variants that may warrant further study and functional analyses. Finally, the comparative analysis of previous genomic studies identified gaps that highlight the need for more expansive analysis of the SMZL genome with unbiased whole genomic approaches.

## Results

### Study selection and characteristics

**Figure 1:**
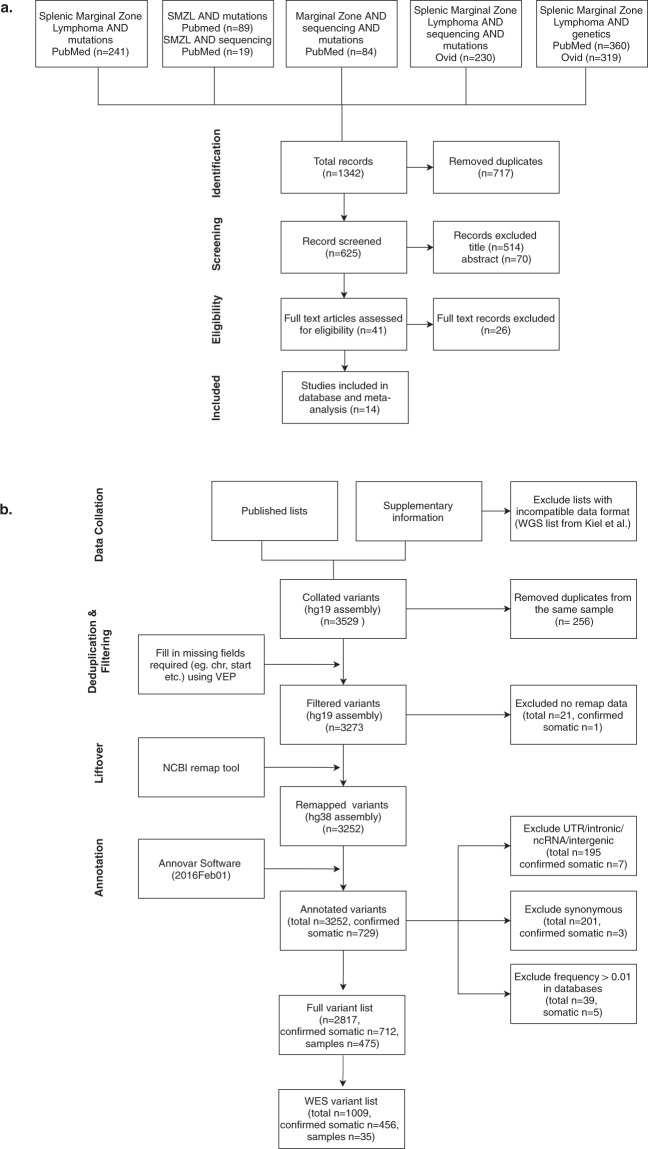
Methods Summary. (**a**) Flowchart of manuscript selection. The figure goes through the search strategy, starting with the combination of search terms used in the databases. Numbers denote amount of records or manuscripts at each step. Once all entries were compiled into a single list, duplicate manuscripts were removed and those remaining were reviewed to identify those that would be used in the full text review. The number of manuscripts excluded and those that were kept are stated in each of the steps. (**b**) Flowchart of database compilation and variant filtering. The flowchart begins at the data collation step, where all the lists of variants from the published manuscripts and Supplementary information were collated into a single list. Subsequent filtering strategies and data manipulation tools are described. Numbers denote amount of variants at each step.

After collating and subsequent removal of duplicate entries, 625 unique manuscripts were retained of the 1342 manuscripts that were initially identified (n = 793 in PubMed, n = 549 in Ovid). After manual inspection of title and abstracts, 584 manuscripts were removed as they did not sequence SMZL cases or were not published in peer-reviewed journals (Supplementary Table S1). For the remaining 41 manuscripts, the full manuscripts and supplementary data were carefully examined to ensure they reported a full list of variants with appropriate sample information and genome mapping details. Twenty-six records were excluded, as they included no list of sequenced variants (Fig. 1a). The 14 remaining studies were split into three types: (1) discovery, (2) confirmation/extension and (3) comparison (Supplementary Table S2). Six discovery genome wide studies^1–6^ implemented WGS or WES with an unbiased approach and subsequently confirmed the variants using targeted sequencing. The nine extension/confirmation studies^1,2,4,6–10,12^ were hypothesis based, targeting pathways identified in discovery cohorts or validation of recurrently mutated genes. Three comparison studies^11,13,14^ sequenced SMZL cases as reference points to compare other B-cell lymphomas. Lack of matched germline allowed only a fraction of the variants in the studies to be confirmed as somatic. The somatic status of each variant and method employed are included in the final list of variants (Supplementary Table S3).

### Database collation

The 3529 variants from 475 cases were extracted from these 14 studies and collated into a single list (Supplementary Table S3). Before further analysis, the variant list was manually curated to exclude the following: a) the supplementary list of WGS variants from Kiel *et al*.^1^ due to format incompatibility and a lack of matched germ-line WGS data; b) 256 variants from the same patients published in two studies based on sample ID and variant characteristics (sequencing depth and variant allele frequency [VAF]); and c) 21 variants due to lack of information necessary to remap and annotate (location and reference/alternate allele) (Fig. 1b). Post-annotation, the following variants were excluded: a) 195 intronic variants; b) 201 synonymous variants and; c) 39 variants with a frequency greater than 1% in known databases of normal variation (Fig. 1b). The majority of the synonymous variants were reported by the Martinez study^4^ (n = 195), as the authors did not remove synonymous variants from the list of total variants published in their supplementary data. After variant filtering the resulting list, termed ‘full variant list’, contained 2817 variants with a subset of 1009 variants resulting from WES, termed ‘WES variant list’, to be used for further analysis. In the ‘full variant list’, 568/2817 were annotated with a COSMIC ID, duplicates included. Figure 1b summarises the filtering criteria and the number of variants removed with each filter. The final number of mutated cases, variants, genes and confirmed somatic variants included in the final database list as well as the WES subset are detailed in Supplementary Tables S4 and S5, respectively.

### Recurrent mutated genes in restricted whole exome sequencing cohort

**Figure 2:**
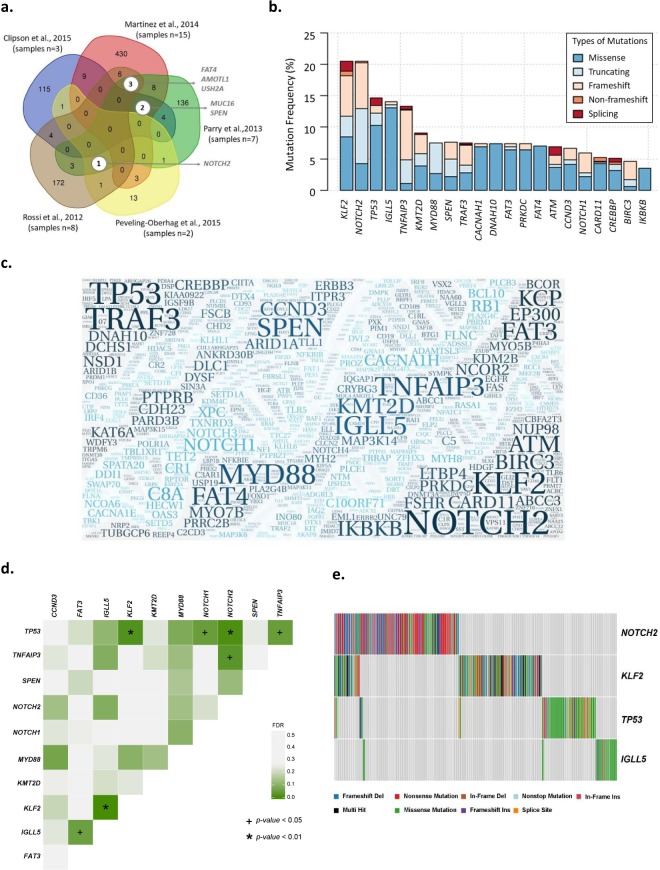
Database analysis. (**a**) Venn diagram of gene overlap in WES studies. The figure shows common and unique genes reported in each study with no overlap between all five. Where there was an overlap of genes identified by more than three studies there are white circles to emphasise the number and name of gene(s) . The gene list was obtained from the WES subset of the final filtered and annotated list. (**b**) Mutation frequency (%) of the top 20 genes. The graph displays the frequency of mutations in each gene as well as an overview of the type of mutations (missense, non-sense, frameshift and splicing). Genes are listed in descending frequency. (**c**) Wordcloud of gene symbols present in database drawn using WordArt (https://wordart.com). The size of each gene symbol is proportional to the number of mutations in each gene (range: 1–123 mutations). *NOTCH2* (n = 123) and *KLF2* (n = 121) had the highest number of mutations, followed by *TNFAIP3* (n = 75), *TP53* (n = 60) and *MYD88* (n = 43). (**d**) DISCOVER mutual exclusivity test results. Heat map displaying the corrected *p*-value for the gene pairs tested for mutual exclusivity in the DISCOVER algorithm. The dark green boxes indicate a low *p-value* where the plus sign (+) those with a *p-value* < 0.05 and the asterisk (*) highlights those pairwise combinations with a *p-value* < 0.01. (**e**) Waterfall plot of mutations found in *KLF2, NOTCH2, TP53* and *IGLL5*. Each column represents a sample, and each row a gene. Each column is coloured according to the mutation type present in the sample and grey if no mutations are present.

Our first task was to perform an unbiased analysis of the genomic landscape of SMZL derived from the ‘WES variant list’, as this allowed an accurate estimation of mutational frequency across the majority of protein-coding genes for all studies. Overall, 35 unique samples were sequenced with WES, accounting for 1009 variants in our final variant list. Figure 2a shows very limited concordance between studies, with no single gene harbouring somatic mutations across all five WES studies. This result further validates the notion that there is a lack of unbiased studies on SMZL and that the current genome-wide datasets are not adequate to fully capture the somatic landscape of SMZL. When we assessed the prevalence of mutations in a given gene across the entire 35 cases, the *SPEN* gene was the most frequently mutated with five somatic variants (5/1009), accounting for 0.5% of the total variants. *FAT4*, *MYD88, NOTCH2*, and *TNFAIP3* all followed with four mutations each. Importantly, the study by Clipson and colleagues was the only study to report KLF2 mutations, a gene of known clinical and biological importance in SMZL pathophysiology, likely missed by other studies as a result of its high GC content.

### Recurrent mutated genes in the total dataset

**Figure 3:**
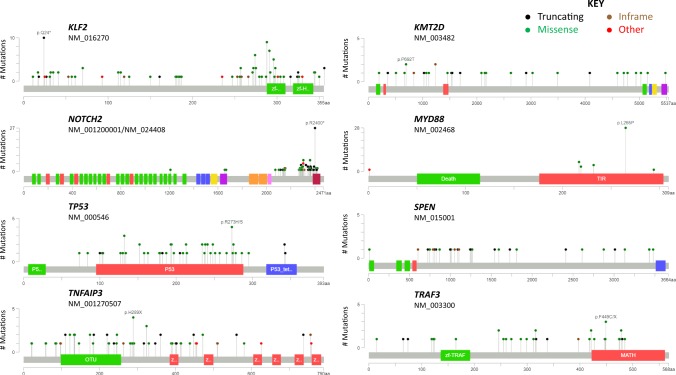
Mutations in key recurrently mutated genes in the database, drawn using cBioPortal (http://www.cbioportal.org/mutation_mapper.jsp). The figure illustrates a linear protein representing each gene with its respective domains. The height is representative of the number of variants reported (The y-axis is not the same proportion for all figures) and circle colour identifies the type of mutation. The transcript used for each protein is stated under the gene name and the colours of the domains was randomly assigned.

Next, we investigated the ‘full variant list’, of 2817 variants in 1239 genes, to define the putative frequency of recurrently mutated genes across all 14 studies. However, one caveat to this analysis is that we had to assume that all genes were screened in all studies, as the total number of genes analysed is not consistently or accurately reported across the targeted re-sequencing studies. This assumption is likely to underestimate the prevalence of mutations in some genes described herein, particularly those that are less well-established, as they were not analysed in all cases. Figure 2b,c show the mutations identified in SMZL and their mutational prevalence. Figure 3 shows the mutations in the eight key genes with the most mutations plotted on a linear protein.

Given the large total cohort included in our systematic review, we could identify recurrent variants in genes of known importance. This is particularly important for future functional validation, as these recurrent somatic variants are prime targets of experimental work. The three most frequently mutated genes, as expected, were *KLF2*, *NOTCH2* and *TP53*. *KLF2* [21%] mutations were found throughout the entire protein and included missense (n = 50), frameshifts (n = 38), truncating (n = 19), splicing (n = 9) and non-frameshift (n = 5). A recurrent *KLF2* variant (p.Q24X) was found in 10 (1.7%, 10/589) cases, that is not annotated in COSMIC, but is predicted to be pathogenic with a CADD phred score of 35^15,16^. *NOTCH2* mutations [20%] clustered in exon 34 that encodes the C-terminal PEST domain (Fig. 3); mutations were truncating (n = 53), frameshifts (n = 44), missense (n = 25), and a single non-frameshift. A recurrent variant (p.R2400X, COSM36210) located in the PEST domain was present in 27 (4.5%, 27/602) cases, predicted to be deleterious with a CADD phred score of 44^15,16^. *TP53* [15%] and *IGLL5* [14%], the third and fourth most frequently mutated genes respectively, harboured mostly nonsynonymous mutations. Mutations in *TP53* were enriched within the DNA binding domain (Fig. 3) where the most recurrent variant (p.R141H) was present in only three of the assessed cases [0.007%]. Twenty eight [28/52] variants were annotated in the IARC *TP53* database^17^.

A number of other genes have previously been shown to be targeted by recurrent mutations, but without concordance across studies. In the ‘full variant list’, *TNFAIP3, KMT2D, MYD88, TRAF3* and *SPEN* were recurrently mutated. Mutations in *TNFAIP3* [13%], *KMT2D* [9%], and *MYD88* [8%] were principally truncating events, where mutations in *TNFAIP3* and *KMT2D* were also distributed throughout the entire gene (Fig. 3). The p.L265P *MYD88* variant accounted for 65% (28/43 mutations) of all *MYD88* variants, is pathogenic according to ClinVar^18^ and annotated in the COSMIC database (COSM85940). This variant is in the toll/interleukin-1 receptor homology (TIR) domain (Fig. 3), and has been shown to be recurrently mutated in a number of mature B-cell tumours^19^. Other recurrent *MYD88* mutations included p.V217F, p.M232T, and p.S219C present in six, four, and three cases respectively. The latter (p.S219C), along with the p.L265P variant, has been identified in a recently recognized entity, termed clonal B-cell lymphocytosis of MZ origin (CBL-MZ)^20,21^, that can sometimes progress to SMZL^22^. The other *MYD88* mutations (p.V217F and p.M232T) have been identified in both CLL and DLBCL^23^. *CCND3* mutations in the C-Terminal domain were mutated in 18 cases (7%). These mutations have been identified in a panel of mature B-cell neoplasms, and most recently in patients with splenic diffuse red pulp small B-cell lymphoma (SDRPL), another entity difficult to differentiate from SMZL^24^. A recurrent variant was also identified in *IKBKB* (p.K169E), reported in eight cases (1.7%, 8/510), that is predicted to be deleterious with a CADD phred score^15,16^ of 29.7 (Supplementary Table S3).

Finally, we attempted to perform some higher-level analysis, particularly pertaining to whether recurrently mutated genes co-exist or are mutually exclusive, extending what has been shown in the literature^2,9,10^. We used the DISCOVER^25^ (Discrete Independence Statistic Controlling for Observations with Varying Event Rates) algorithm to test for associations of co-occurrence and mutual exclusivity between genes in the database. This independent test takes into account the overall alteration rates of each individual tumour by creating a background matrix which is how tumour specific alteration rates are incorporated by the test. The background matrix is created with simple binary mutation matrix of m*n dimension where ***m*** is the number of genes and ***n*** the number of cases to get a genome wide view of each tumour. Studies (n = 6) that that did not assess more than 100 genes were excluded from this analysis to reduce bias, leaving 240 cases to be assessed. Furthermore, only genes that were mutated in at least 15 tumours were used in the pairwise test leaving 11 genes to be assessed. The pairwise combinations that were significantly mutually exclusive were: 1) *KLF2* and *IGLL5* with the highest significance of mutual exclusivity (*p* = 0.001); 2) *TP53* and *NOTCH2* (*p* = 0.008) and; 3) *TP53* and *KLF2* (*p* = 0.01). Figure 2d shows a heat map of the corrected *p*-values for the pairwise combinations and Fig. 2e a waterfall plot of *KLF2, NOTCH2, TP53* and *IGLL5* where the mutually exclusive genes pairs can be better visualised. We did not perform an analysis of clonal evolution, as limited VAF information was available for key genes, such as for *KLF2* and *NOTCH2* variants, as they have principally been identified with Sanger sequencing. Therefore, we could not meaningfully extended the findings of Parry and co-workers^9^.

## Discussion

Currently, there is a scarcity of genome-wide studies that document the somatic landscape of SMZL. The 35 published cases with WES or WGS data available provide only an incomplete catalogue of the genes and pathways disrupted in the disease, failing to recapitulate the clinical heterogeneity that defines SMZL. Therefore, we chose to perform a systematic review of mutational data from fourteen studies covering three broad categories; 1) discovery WES/WGS studies of SMZL; 2) confirmation/extension of SMZL usually utilizing targeted re-sequencing approaches; and 3) sequencing data drawn from studies of other mature B-cell neoplasm, where SMZL was included as comparison. Our aims were to: a) determine if a systematic review approach would yield novel insight into the genes and pathways targeted by somatic mutations; and b) provide the research community with a unique and accurately annotated database of SMZL mutations, mapped to functionally relevant transcripts in GRch38 to facilitate future investigations.

We encountered a number of limitations that restricted the scope of our analysis. These issues are broadly relevant to any systematic review approaches, pertaining to experimental/analytical designs and the lack of all the required information in published studies. For example, Parry *et al*. provided a detailed list of the somatic variants detected in their study, with expansive annotation, whilst Clipson and co-workers published only high-level amino-acid sequences. Furthermore, it was not possible to consistently account for variability associated with the nature of the original tumour samples themselves, such as tumour cell purity, and as raw sequencing data was not reported with the data, we could not accurately account for sequence coverage or read depth, nor could we account for potential bias originating from the bioinformatics analysis. As our combined analysis included WES and re-sequencing studies, the overlap in those genes targeted for analysis was inconsistent. This introduced certain biases towards genes included in the panel design of a specific study, which would result in more compelling data emerging from genes included in more studies, due to their putative role in SMZL or aligned conditions.

WES/WGS studies are often limited by sample size and this is certainly the case for SMZL. According to the ICGC, WES/WGS analysis of 500 tumour/normal duos are needed to reliably detect genes that are somatically mutated in 3% of tumour^26^. For rarer tumours, they propose a two-tiered strategy to obtain comparable statistic power processing 100 discovery cases with validation of all mutated genes in an additional 400 cases. The putative power of these approaches will vary based on the mutational burden of a specific tumour type, the background mutation rate, and variability in sequencing depth^26^. Whilst 14 studies were selected for our systematic review, only the five WES studies, including 35 patients, were used to perform an unbiased analysis of all coding genes, far below the cohort sizes proposed by the ICGC. This is the likely explanation for the restricted concordance we observed between WES studies (Fig. 2a), supporting our view that the current genome-wide datasets are inadequate to accurately capture the SMZL genome.

In spite of these limitations, we were able to provide the following insights. Firstly, we confirm the fundamental importance of *NOTCH2*, *KLF2* and *TP53* in a pathogenesis of SMZL. *NOTCH2* mutations targeted the C-terminal PEST domain necessary for the regulation of the intracellular domain (NICD)^27^, and consequent transcriptional regulation. Two distinct clusters of mutations were found in *KLF2*, one consists mostly of missense mutations flanking the ZF1 domain involved in DNA recognition, and the second in the activation domain. Several of these mutations, particularly those in the first cluster have been shown to hinder the ability of *KLF2* to suppress NF-κb induction by upstream signalling pathways^6^. Whilst *KLF2* mutations have been shown to be associated with other clinic-biological features of the disease (del7q and IGHV1–2*04 usage)^6,10^, we could not extend these findings, as additional patient information was not available on many of the cases. *TP53* was recurrently mutated in our analysis, supporting the critical role this gene plays in cancer and more specifically in SMZL. As is observed in other mature B-cell tumours, mutations were clustered in the DNA binding domain, where they lead to protein dysfunction.

One advantage of our approach was that we could provide greater granularity on the importance of genes that are mutated in any single study at lower prevalence. In our study we confirm the importance of genes in the NF-κb pathway; specifically *TNFAIP3 (13%)*, MYD88 (8%), *TRAF3* (8%), *CARD11* (5%), *IKBKB* (4%), and *BIRC3* (4%). Notably *TRAF3* was mutated across studies, and warrants further analysis at the molecular and functional level. *KMT2D* is mutated in FL and DLBCL, where it functions as a tumour suppressor, promoting lymphomagenesis in murine models^28^. In our full variant list, *KMT2D* was mutated in 9% of cases. Recurrent *IGLL5* mutations have been identified in CLL, linked to canonical activation induced-cytidine deaminase (AID) activity with a mutation pattern clustering around the transcription start site within the first intron^29^. AID induces clustered mutations in the immunoglobulin loci as well as some off target regions, potentially an underlying cause of oncogenic mutations often seen in B-cell malignancies^30^. *IGLL5* is homologous to *IGLL1*, critical for B-cell development and hints at having a biological importance in CLL^29^. In our pooled dataset, we found *IGLL5* mutations in 31/222 cases (14%), clustering around the first exon similar to *IGLL5* mutations observed by Kasar and colleagues in CLL cohort^29^. *IGLL5* mutations have also been reported in other marginal zone (nodal and extranodal) and lympho-plasmacytic lymphomas. Pillonel *et al*. (2018) reported *IGLL5* to be the most frequently mutated gene in SMZL along with *NOTCH2* where both genes had a 25% mutational frequency in SMZL cases^14^. Although we do not have sufficient sequencing information to determine the mutational signature underpinning *IGLL5*, it is likely similar to the situation in CLL, where it has a high mutational frequency as consequence of off-target AID activity. Another advantage of our analysis is that we were able to look at the co-existence of somatic mutations in a larger cohort of SMZL cases. The DISCOVER algorithm we employed identified three pairs of genes (*KLF2* and *IGLL5, TP53* and *NOTCH2*, and *TP53* and *KLF2*) which were significantly mutually exclusive, suggesting possible disease subtypes in SMZL. It was not possible to do any further correlations with these results as the individual phenotypes were not published with the manuscripts.

Finally, and perhaps most importantly, this endeavour represents a critical community database as currently SMZL tumours are not included in the IGCG and TCGA, and only 23% of reported SMZL variants are included in COSMIC. This is illustrated by the current status of the COSMIC database that omits recurrent mutations in *KLF2*, the most important recurrently mutated gene in SMZL, supported by molecular and cellular experimental work^6,9,10^. In essence, this means that presently accessible resources cannot support the interpretation of SMZL-specific somatic mutations. This emphasises the importance of this resource, which is fulfilling a currently unmet need, permitting scientists to more accurately interpret somatic mutational data in SMZL. Indeed, our study is an exemplar for all rare tumour types, where similar resources would be valuable.

In conclusion, we provide unequivocal evidence that the study of SMZL genomics requires expansive unbiased whole genome mutational analysis to fully unravel the somatic landscape of the disease. Indeed, WGS will be required to ascertain the importance of non-coding mutations, and the presence of key mutational mechanisms. These approaches will provide a more methodical view of the disease, permitting these observations to be more successfully implemented into clinical care. Our systematic review confirms the importance of *NOTCH2, KLF2* and *TP53*, and adds evidence to the importance of a number of other genes, such as *TNFAIP3*, *TRAF3*, and *KMT2D*, that will guide future molecular screening and functional experimentation. Whilst this study represents an important contribution to our understanding of SMZL, future genomic analysis is required that will ultimately be linked to transcriptomic, epigenomic and proteomic datasets, to fully elucidate the molecular pathogenies of SMZL. This will be supported by the analysis of consistently treated patients entering clinical trials of immune-chemotherapy and targeted agents. The hope is that collectively, these approaches with benefit the management of patients will SMZL, predicting patient’s survival time, risk of transformation and enable more accurate diagnosis to distinguish SMZL from other mature B-cell lymphomas.

## Methods

### Search strategies and study selection

This study was performed according to the principles of the PRISMA-P Preferred Reporting Items for Systematic Review and Meta-analysis Protocol^31–33^. The literature search was undertaken by two investigators independently in January 2019 using Ovid (http://ovidsp.ovid.com) and PubMed (https://www.ncbi.nlm.nih.gov/pubmed) as the primary search engines. The keywords that were used included: “Splenic Marginal Zone Lymphoma”, “SMZL”, “Marginal Zone”, “sequencing”, “genetics” and “mutation”. Two independent investigators performed data collation, duplicate entries were removed, and manuscript titles and abstracts were reviewed to identify those that would be used in the full text review. Studies for inclusion were defined using the following steps; (1) titles were scanned for those that included MPS or Sanger sequencing of SMZL or other mature B-cell lymphomas; (2) the abstracts from these manuscripts were examined to identify studies that included SMZL cases with a confirmed immunophenotypic or pathological diagnosis; (3) the full text and supplementary data were assessed to select studies that reported a full list of variants with appropriate sample information and genome mapping details. Studies that did not meet these criteria were assessed in detail and excluded from future analysis. Our search was limited to studies written in English.

### Data extraction

Genomic information was extracted from the published studies, from both the main manuscript and supplementary material. The final list of variants was assembled in an excel document where missing base pair location and reference and alternate allele information was filled with the GRCh37 assembly of Ensemble Variant Effect Predictor (VEP)^34^ (http://grch37.ensembl.org/Homo_sapiens/Tools/VEP) using the mutated gene and nucleotide change reported. It should be noted that for each variant VEP will output several transcripts each with a different base pair location. To overcome inconsistencies, three transcript tags (transcript support level, APRIS and GENCODE Basic) present in the VEP annotation were inspected to identify the highest quality and most relevant transcript. Variants from the same sample that appeared in more than one study were flagged using the sample ID, location and protein change and were included only once following manual curation. Once the list was populated, variants with insufficient data for remapping were removed due to uncertainty, across studies, of transcript used for reporting variants. The remaining variants were remapped from GRCh37 to the GRch38 genome assembly with the NCBI remap tool (https://www.ncbi.nlm.nih.gov/genome/tools/remap). Finally, the remapped list of variants was annotated using the Annovar software (2016Feb01)^35^ adding gene-based annotation to identify functional effects, frequency of the variant in specific databases, and scores that predict how mutations affect protein function. Additional information found in the manuscripts and supplementary information was also added which included: VAF; depth; confirmed somatic status (lack of variant in match germ-line DNA); sequencing method by which somatic status was confirmed and sequencing approach. Preceding data analysis, the variant list was filtered to retain only those variants that are likely to be somatic mutations or likely drivers of disease. Variants that were filtered out were those that: a) fell within UTRs and intergenic regions; b) synonymous variants and; c) variants that had a frequency greater than 1% in databases of normal germline variation (The Genome Aggregation Database^36^, 1000 Genomes Project^37^, NHLBI GO Exome Sequencing Project, Exome Aggregation Consortium^38^). The final database comprised all of the remaining variants and these were the focus of subsequent analysis. Variants resulting from WES studies were evaluated separately.

## Supplementary information

Supplementary Tables

PRISMA checklist
